# LRRC8A promotes *Glaesserella parasuis* cytolethal distending toxin-induced p53-dependent apoptosis in NPTr cells

**DOI:** 10.1080/21505594.2023.2287339

**Published:** 2023-11-29

**Authors:** Weiting Mao, Zhichao Wang, Siting Wen, Yan Lin, Jiayun Gu, Ju Sun, Huan Wang, Qi Cao, Yindi Xu, Xiaojuan Xu, Xuwang Cai

**Affiliations:** a National Key Laboratory of Agricultural Microbiology, College of Veterinary Medicine, Huazhong Agricultural University, Wuhan, China; b Key Laboratory of Preventive Veterinary Medicine in Hubei Province, Cooperative Innovation Center for Sustainable Pig Production, Wuhan, China; c Institute of Animal Husbandry and Veterinary Research, Henan Academy of Agricultural Sciences, Zhengzhou, China

**Keywords:** Glaesserella parasuis, cytolethal distending toxin, cell apoptosis, p53 signalling, LRRC8A

## Abstract

*Glaesserella parasuis* is an early colonizer of the swine upper respiratory tract and can break through the respiratory barrier for further invasion. However, the mechanisms underlying *G. parasuis* increases epithelial barrier permeability remain unclear. This study demonstrates that *G. parasuis* cytolethal distending toxin (CDT) induces p53-dependent apoptosis in new-born piglet tracheal (NPTr) cells. Moreover, we report for the first time that leucine-rich repeat-containing protein 8A (LRRC8A), an essential subunit of the volume-regulated anion channel (VRAC), involves in apoptosis of NPTr cells mediated by *G. parasuis* CDT. Pharmacological inhibition of VRAC with either PPQ-102 or NS3728 largely attenuated CDT-induced apoptosis in NPTr cells. Additionally, experiments with cells knocked down for LRRC8A using small interfering ribonucleic acid (siRNA) or knocked out LRRC8A using CRISPR/Cas9 technology showed a significant reduction in CDT-induced apoptosis. Conversely, re-expression of *Sus scrofa* LRRC8A in *LRRC8A*^–*/*–^ NPTr cells efficiently complemented the CDT-induced apoptosis. In summary, these findings suggest that LRRC8A is pivotal for *G. parasuis* CDT-induced apoptosis, providing novel insights into the mechanism of apoptosis caused by CDT.

## Introduction

*Glaesserella parasuis* (formerly known as *Haemophilus parasuis*) is a commensal organism of the upper respiratory tract of swine and is the aetiological agent of Glässer’s disease, characterized by fibrous polyserositis, meningitis, and arthritis [1]. However, the molecular mechanisms underlying *G. parasuis* interactions with host cells are largely unknown, which makes it challenging to prevent and control the disease.

Cytolethal distending toxin (CDT) is a significant element in Glässer’s disease. CDT is a bacterial exotoxin secreted by several Gram-negative pathogenic bacteria such as *Actinobacillus actinomycetemcomitans* [2], *Escherichia coli* [3], *Haemophilus ducreyi* [4,5], *Campylobacter* spp. (*C. jejuni*, *C. fetus*, *C. coli*, *C. upsaliensis*, *C. hyointestinalis*, and *C. lari*) [6], and *Providencia alcalifaciens* [7]. Generally, the pathogenicity of CDT is reflected in two aspects. On the one hand, CDT is capable of inducing apoptosis in B and T lymphocytes to downregulate the host immune response, allowing bacterial proliferation and causing more tissue damage [8]. In contrast, the ability of CDT to induce cell death results in increased epithelial barrier permeability, which enables bacterial invasion [9]. A complete CDT consists of three subunits: CdtA, CdtB, and CdtC [10]. CdtA and CdtC mainly bind to lipid rafts, which is a mechanism responsible for the translocation of the catalytic subunit CdtB into cells. CdtB entering the cell acts as a nuclease similar to DNase I and ultimately leads to irreversible cell cycle arrest and cell apoptosis through DNA damage [11–13]. However, the effect of *G. parasuis* CDT on the respiratory tract has not been studied.

The p53 signalling pathway is essential for the regulation of cell cycle progression, DNA damage response, and apoptosis [14]. In some abnormal cellular conditions, such as DNA damage, p53 protein binds to the corresponding site of the gene and exerts its effect as a special transcription factor to induce cell cycle arrest, which would allow sufficient time to repair DNA or induce apoptosis if repaired unsuccessfully [11,15]. Two distinct pathways, the death receptor pathway and the mitochondrial pathway, can lead to apoptosis [16]. In the mitochondrial pathway, the anti-apoptotic protein B cell lymphoma 2 (Bcl-2), located in the outer mitochondrial membrane *via* heterodimerization with the pro-apoptotic protein Bcl-2-associated X (BAX), controls apoptosis [17]. Notably, p53-dependent apoptosis involves the mitochondrial pathway, including the neutralization of Bcl-2, release of BAX, mitochondrial outer membrane permeabilization (MOMP), and activation of caspase-9 and caspase-3 [18].

A pivotal standpoint is that apoptosis is generally accompanied by volume changes that represent the flow of intracellular ions [19]. Extensive research has shown that apoptosis volume decrease (AVD), involving the volume-regulated anion channel (VRAC), is a prerequisite for apoptotic events and results in cell contraction at the onset of apoptosis [20,21]. VRAC is ubiquitously expressed in almost all types of vertebrate cells and is the key player in regulating cell volume by mediating the efflux of Cl^–^ and some organic osmolytes such as taurine [22]. It is now believed that leucine-rich repeat-containing protein 8A (LRRC8A), also named SWELL1, is the only mandatory subunit of VRAC, but it has to combine with at least another LRRC8 protein (LRRC8B – E) to produce a functional channel complex [23–25]. In addition to cell volume regulation and participation in apoptosis, LRRC8A has also been associated with cell proliferation, oxidative stress, insulin secretion by β cells, and anti-viral immunity [26–29]. With respect to the role of LRRC8A in apoptosis, previous studies have focused on the resistance of cells to anticancer drugs such as cisplatin and temozolomide, which induce apoptosis *via* DNA damage [30–33]. However, it remains unclear whether LRRC8A functions in the process of apoptosis induced by bacterial CDT, which causes DNA damage similar to cisplatin and temozolomide.

In the present study, we investigated the effect of *G. parasuis* CDT on new-born piglet tracheal (NPTr) cells and the potential role of LRRC8A in this process. Our study supports the notion that LRRC8A plays a central role in *G. parasuis* CDT-induced apoptosis and provides new insights into its function as an effector or biomarker of apoptosis.

## Materials and methods

### Plasmids, antibodies and reagents

For recombinant expression in *E. coli* BL21 (DE3), *cdtA, cdtB*, and *cdtC* of *G. parasuis* were inserted into the pET-30a plasmid encoding an N-terminal His_6_-tag. For stable expression of LRRC8A, the sequence of *LRRC8A* was inserted into the pcDNA3.1 vector and transformed into *LRRC8A*^–*/*–^ NPTr cells. All the plasmids used in this study were verified by DNA sequencing. The bacterial strains and plasmids used are listed in Table 1.

**Table 1.** Bacterial strains and plasmids used in this study.

Strain or plasmid	Genotype and/or phenotype	Reference or source
*E. coli* DH5α	A cloning vector: F^−^, φ80*lacZ*ΔM15Δ (*lacZY*A-*argF*) U169, *deoR*, *recA1*, *endA1*, *hsdR17* (rk^−^, mk^+^), *phoA*, *supE44*, λ^−^, *thi-*1, *gyrA96*, *relA*1	TaKaRa Bio Inc., Dalian, China
*E. coli* BL21 (DE3)	F^−^, *ompT*, *hsdS*_*B*_ (r_B_^−^m_B_^−^), *gal*, *dcm* (DE3)	Invitrogen, Carlsbad, CA, USA
pET30a	Expression vector; Kan^r^	Novagen, Madison, WI, USA
pET-30a*cdtA/B/C*	Genomic sequence available	This laboratory
YSY-spCas9-Puro	CRISPR/Cas9 KO vector, Puro^r^	YSY biotech, Nanjing, China
pcDNA3.1	Expression vector; Amp^r^	Invitrogen, Carlsbad, CA, USA

Kan^r^, kanamycin-resistance; Amp^r^, ampicillin-resistance.

Antibodies against phosphorylated p53 (p-p53), p53, and His_6_-tag were obtained from ABclonal (Wuhan, Hubei, China), and antibodies against Bcl-2, BAX, and Glyceraldehyde-3-phosphate dehydrogenase (GAPDH) were purchased from Proteintech (Wuhan, Hubei, China). The antibody against LRRC8A was obtained from Abcam (Cambridge, UK).

The reagents were purchased as follows: Annexin V-fluorescein isothiocyanate (Annexin V-FITC)/propidium iodide (PI) Kit from 4A Biotech (Beijing, China); VRAC inhibitors PPQ-102 and NS3728 from MedChemExpress (Monmouth Junction, NJ, USA), and GreenNuc^TM^ Caspase-3 Assay Kit for Live Cells from Beyotime (Shanghai, China). Unless otherwise noted, all other chemicals and reagents were purchased from Sigma-Aldrich (St. Louis, MO, USA).

### Cell culture and treatment

A new-born piglet tracheal (NPTr) cell line was obtained based on continuous culture of primary cells derived from tracheal tissues [34]. NPTr cells were cultured in Dulbecco’s Modified Eagle’s Medium (DMEM)-high glucose supplemented with 10% foetal bovine serum (FBS) (Gibco, Grand Island, NY, USA). All cells were maintained at 37°C in an incubator containing 5% CO_2_ and tested to be *Mycoplasma*-negative for mycoplasma using the standard PCR method [35].

Cells were pre-seeded overnight in 6-well plates (1 × 10^5^ cells) until 60% confluence was reached the next day. Different doses of CDT were added to the culture medium. Relevant experiments were conducted after 36 h. To inhibit the activity of VRAC, cells were pre-treated with either the VRAC inhibitor PPQ-102 or NS3728 for 2 h before supplementation with CDT. The final doses were 500 ng/mL for CDT, 30 μM for PPQ-102, and 10 μM for NS3728.

### Immunofluorescence staining

Cells grown on confocal dishes were treated with 100 MOI live bacteria or 500 ng/mL CDT for 36 h. Cells were fixed in 4% paraformaldehyde for 30 min. The cells were incubated with 0.5% Triton X-100 in phosphate-buffered saline (PBS) for 15 min. After blocking in 5% bovine serum for 1 h at room temperature, the cells were incubated with rabbit anti γH2AX monoclonal antibody (Cell signalling technology, Denver, MA, USA) at 4°C overnight. The confocal dishes were washed and incubated with Cy3-conjugated secondary antibodies for 1 h and DAPI for 15 min. Anti-fade solution was added to the confocal dishes and the confocal dishes were visualized using a Zeiss LSM 510 META fluorescence microscope (Oberkochen, Baden-Württemberg, Germany).

### Cell viability and lactate dehydrogenase release assay

Cells were seeded in 96-well plates. Then stimulated with 100 MOI of live bacteria or 500 ng/mL CDT for 36 h. Cell viability was measured by cell counting kit-8 (CCK-8) assay (Yeasen, Shanghai, China), and lactate dehydrogenase (LDH) was measured using LDH cytotoxicity assay kit (Beyotime, Shanghai, China). These experiments were performed according to the manufacturer’s protocol and repeated three times.

### Expression and purification of recombinant proteins

To obtain *G. parasuis* CdtA, CdtB, and CdtC recombinant proteins, *E. coli* BL21 (DE3) harbouring the expression vector was cultured at 37°C and induced by adding isopropyl-β-D-thiogalactopyranoside (IPTG) to a final concentration of 0.1 mM when OD_600 nm_ reached 0.5–0.6, followed by incubation at 37°C for 8 h. A total of 400 mL bacterial cultures were harvested and subsequently resuspended in 40 mL buffer A (50 mM Tris-base, 0.5 mM EDTA, 50 mM NaCl, pH 8.0), and disrupted by sonication at 400 W for 5 min (Sonics, Newtown, CT, USA). Inclusion bodies were isolated by centrifugation at 6,000 × *g* for 30 min at 4°C and washed three times with 40 mL of buffer A. The washed inclusion bodies were solubilized in 40 mL buffer A supplemented with 5 mM dithiothreitol (DTT) and 750 mg/mL sodium lauroylsarcosinate (SKL) at 4°C for 12 h. Then, the precipitates were isolated by centrifugation at 6,000 × *g* for 30 min at 4°C, followed by the addition of 800 μL refolding buffer (20% PEG4000, 100 mM reduced glutathione, 50 mM oxidized glutathione) at 4°C for 2 h. The samples were dialysed against 1 × TE buffer (pH 8.0) at 4°C for 72 h. N-terminal His_6_-tagged CdtA, CdtB, and CdtC proteins were purified by affinity chromatography using Ni Sepharose^TM^ 6 Fast Flow (GE Healthcare Life Science, Pittsburgh, PA, USA). Purified recombinant proteins were confirmed by SDS-PAGE and Western blot analyses. The three subunits were reconstituted in complete CDT at 25°C for 1 h for subsequent experiments.

### Flow cytometry

Cells were pre-seeded in 6-well plates (1 × 10^5^ cells) for 12 h and treated with different doses (0, 5, 50, or 500 ng/mL) of CDT for 36 h. After collection and washing twice with cold PBS, the cells were stained using the Annexin V-FITC/PI Apoptosis Assay Kit (4A Biotech, Beijing, China), according to the manufacturer’s instructions. Stained cells were analysed with a BD FACSVerse^TM^ flow cytometer (BD Biosciences, San Jose, CA, USA), and data were processed using FlowJo software (Ashland, Oregon, USA).

### qRT-PCR

Total RNA was extracted using the TRIzol reagent (Invitrogen, Carlsbad, CA, USA). The isolated mRNA was converted to cDNA using a Reverse Transcription Kit (Vazyme Biotech, Nanjing, Jiangsu, China) according to the manufacturer’s instructions. The qRT-PCR reactions were performed using 2 × AceQ® qPCR SYBR® Green Master Mix (Vazyme Biotech, Nanjing, Jiangsu, China) and appropriate primers (Table 2). The expression levels of the target genes were normalized using GAPDH as an internal reference and were calculated using the 2^–ΔΔCt^ method [36]. Data are presented as mean ± SD from three independent assays.

**Table 2.** Gene-specific primers used in this study.

Gene	Primer designation	Nucleotide sequence (5ʹ–3ʹ)	Size of PCR product (bp)	GenBank accession number
*GAPDH*	GAPDH-F	CACAGTCAAGGCGGAGAAC	106	NM_001206359.1
	GAPDH-R	CGTAGCACCAGCATCAC		
*BAX*	BAX-F	TTTGCTTCAGGGTTTCATCCA	114	XM_003127290.5
	BAX-R	AGACACTCGCTCAACTTCTTG		
*P53*	p53-F	CACGAACTGGCTGGATGA	170	NM_213824.3
	P53-R	GGAACCCTAGACGGAAATCA		
*Bcl-2*	Bcl-2-F	GCAGGTATTGAACGAACTC	103	NM_214285.1
	Bcl-2-R	CTCCTTGTCTACGCTCTC		
*LRRC8A*	LRRC8A-F	CCCAAGCTTATGATTCCGGTGACGGAGC	2,430	XM_001928850.6
	LRRC8A-R	CCGGAATTCGGCCTGCTCCTTGTCG		

### Western blot

Cells were lysed by cell lysis buffer for Western blot (Beyotime, Shanghai, China) and then separated by SDS-PAGE (12.5%). The separated proteins were transferred to 0.45 μm polyvinylidene fluoride (PVDF) membranes (Millipore, Billerica, MA, USA), followed by blocking in 5% skimmed milk for 1 h at room temperature. The membranes were incubated with antibodies targeting p53, p-p53, BAX, Bcl-2, GAPDH, or LRRC8A at a dilution of 1:1,000 at 4°C overnight and then incubated with HRP-conjugated secondary antibodies at room temperature for 2 h. The results of Western blot were densitometrically quantified and analysed using the ImageJ software (Bethesda, MD, United States, USA).

### Caspase-3 activity assay

Caspase-3 activity was determined by the cleavage of N-Acetyl-Asp-Glu-Val-Asp *p*-nitroanilide (Ac-DEVD-*p*NA) to produce yellow *p*-nitroaniline (*p*NA), and the fluorescence intensity was measured at 405 nm for excitation. Caspase-3 activity was measured using a Caspase-3 Activity Assay Kit (Beyotime, Shanghai, China) according to the manufacturer’s instructions.

### CRISPR/Cas9-mediated knockout and siRNA-mediated knockdown

In this study, CRISPR/Cas9 technology was used to generate LRRC8A-knockout cell lines. In our experiment, two guide RNAs (gRNA), 5ʹ-CACCGTCCTGCAACGACTCGTTCCG-3ʹ and 5ʹ-CACCGTGCCCGTAGTTCATGCTGGA-3ʹ to target LRRC8A, were respectively cloned into the gRNA-expression plasmid pCMV-Cas9-Puro (YSY Biotech, Nanjing, Jiangsu, China) before the protospacer adjacent motif (PAM). Two micrograms of pCMV-Cas9-Puro plasmid was transfected into 1.5 × 10^6^ NPTr cells using the JetPRIME® Versatile DNA Transfection Reagent (Polyplus-transfection, Illkirch, Strasbourg, France) according to the manufacturer’s instructions. Cells were selected with 2.5 μg/mL puromycin for approximately 5–7 days after transfection for 48 h. The gRNA-expressing cells were trypsinized, counted, and cultured in 96-well plates at a density of approximately one cell per well. Single clones were then cultured for 10–14 days or longer. The correct construction of the knockout cell lines was confirmed by DNA sequencing.

For siRNA knockdown, two specific siRNAs (5ʹ-GGUACAACCACAUCGCCUA-3ʹ and 5ʹ-AGUACGACCUGGACCGACA-3ʹ) (GenePharma, Wuhan, Hubei, China) were used to transiently knockdown LRRC8A expression; the sequences were designed according to a previous study [25]. The 25 μM LRRC8A siRNA and scramble siRNA were transfected into 6-well plates (1.5 × 10^6^ NPTr cells) using the JetPRIME® Versatile siRNA Transfection Reagent (Polyplus-transfection, Illkirch, Strasbourg, France), according to the manufacturer’s instructions. Twenty-four hours after transfection, the cells were used for further experiments, and the knockdown efficiency of *LRRC8A* was measured using qRT-PCR.

### Statistical analysis

Data were compared by one-way or two-way ANOVA using GraphPad Prism version 8.0 (San Diego, CA, United States). All results are expressed as the mean ± SEM from at least three replicates for each experiment. *P < 0.05, considered statistically significant.

## Results

### *G. parasuis* CDT is cytotoxic to NPTr cells

**Figure 1.**
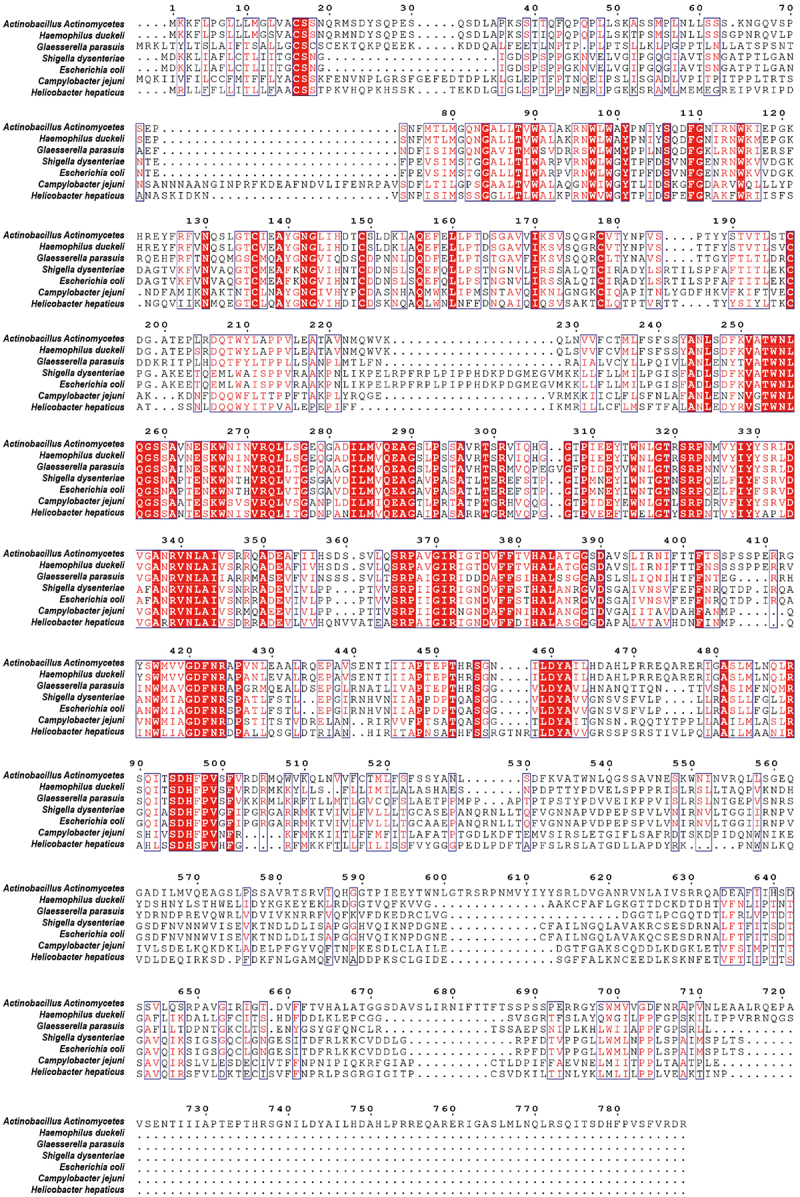
Sequence alignment between CDT holotoxin from *A. actinomycetemcomitans, H. ducreyi, G. parasuis, S. dysenteriae, E. coli, C. jejuni*, and *H. hepaticus*. the alignment was generated using ClustalW2 algorithm and presented using ESPript 3.0 (http://espript.Ibcp.fr/ESPript/cgibin/ESPript.Cgi). Identical residues are indicated by the dark red background and conserved residues are in red text.

**Figure 2.**
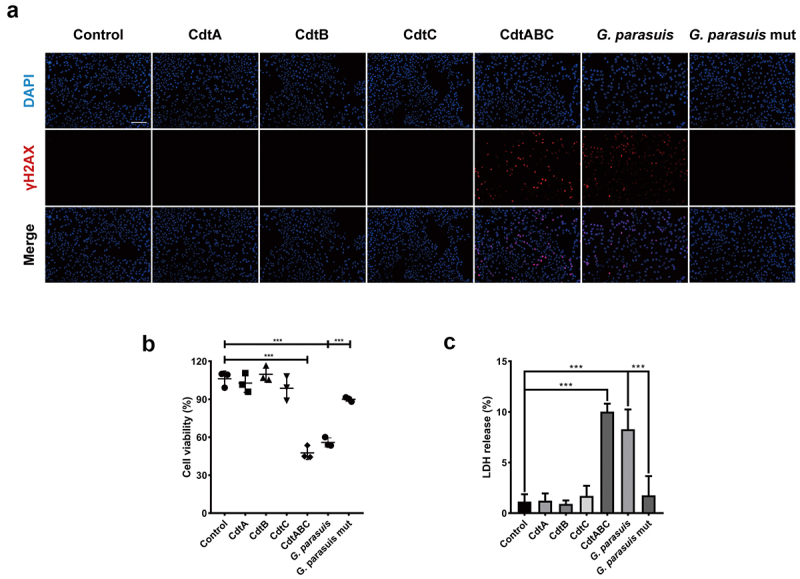
*G. parasuis* CdtABC induces DNA double-strand breaks (DSB), cell death and inhibits cell viability in NPTr cells. NPTr cells were treated with 500 ng/mL *G. parasuis* CdtA, CdtB, CdtC, CdtABC, 100 MOI of live *G. parasuis* WT strain (*G. parasuis*), and 100 MOI of live *G. parasuis* mutant strain (*G. parasuis* mut) for 36 h. (A) DNA damage marker γH2AX was measured using immunofluorescence microscopy. Scale bar 100 μm. (B, C) after treated with *G. parasuis* CdtA, CdtB, CdtC, CdtABC, live *G. parasuis* WT strain, and live *G. parasuis* mutant strain, cell viability and LDH release were measured using CCK-8 and LDH release assay (*n* = 3).

The CDT secreted by *G. parasuis*, which is an opportunistic pathogen, is less conserved than CDTs secreted by other pathogenic bacteria, with amino acid identities of 30.92%, 32.78%, 32.92%, 33.76%, 38.1%, and 48.8% in *C. jejuni*, *Shigella dysenteriae*, *E. coli*, *Helicobacter hepaticus*, *A. actinomycetemcomitans*, and *H. ducreyi*, respectively (Figure 1). Although CDT produced by several pathogenic bacteria has been proven to induce apoptosis in target cells, it is not clear how they interact with epithelial cells to break through the respiratory barrier. To investigate the cytotoxicity of *G. parasuis* CDT in NPTr cells, three CDT subunits, CdtA, CdtB, and CdtC, were expressed and purified. NPTr cells were then stimulated with recombinant proteins CdtA, CdtB, CdtC, holotoxin CdtABC (500 ng/mL), 100 MOI of live *G. parasuis* wild-type (WT) strain, and 100 MOI of live CDT-deficient mutant strain of *G. parasuis* for 36 h. As shown in Figure 2a, CdtABC and *G. parasuis* WT strain treatments increased the level of γH2AX, a DNA damage marker, in NPTr cells, when compared to individual subunit and *G. parasuis* mutant strain treatments. Additionally, CCK-8 and LDH release assays were performed to evaluate the cytotoxicity of *G. parasuis* CdtABC in NPTr cells. The results showed that cell viability was attenuated and the release of LDH was significantly increased in NPTr cells exposed to CdtABC and *G. parasuis* WT strain when compared to individual subunits and *G. parasuis* mutant strain (Figure 2b,c). Collectively, these data reveal that *G. parasuis* CdtABC is cytotoxic to NPTr cells.

### *G. parasuis* CDT induces apoptosis in NPTr cells via p53-dependent pathway

**Figure 3.**
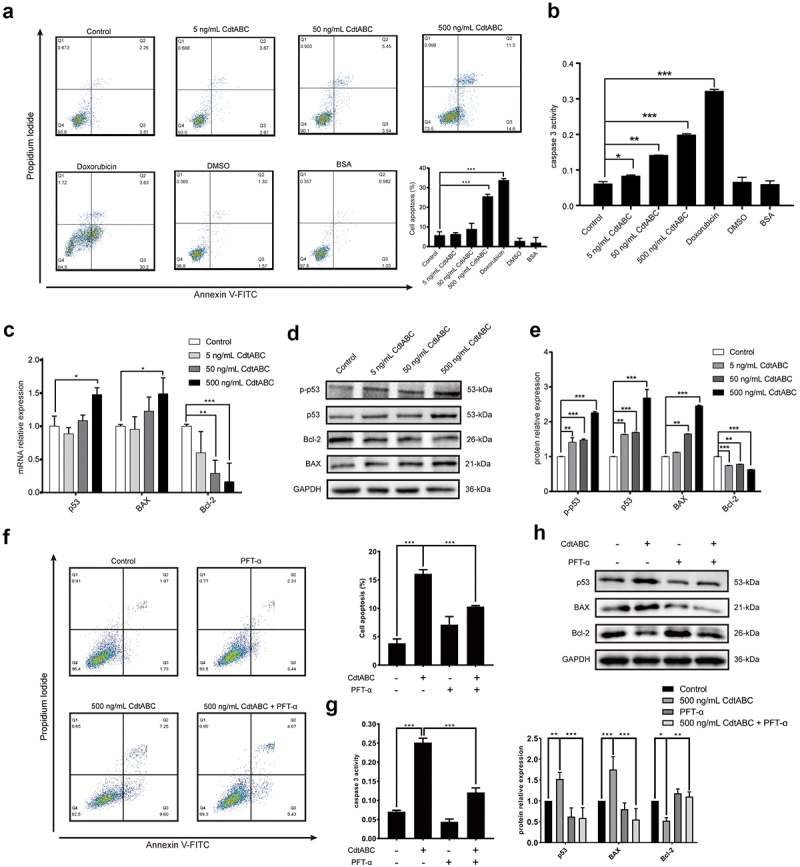
*G. parasuis* CdtABC induces apoptosis in NPTr cells *via* p53-dependent pathway. NPTr cells were treated with different concentrations (0, 5, 50, or 500 ng/mL) of *G. parasuis* CdtABC for 36 h. (a) the percentage of apoptotic cells in NPTr cells was measured using flow cytometry. The doxorubicin was used as a positive control, while DMSO and BSA were used as negative controls. (b) activity of apoptosis factor caspase-3 was measured in NPTr cells. (c) the mRNA levels of p53, BAX, and Bcl-2 in NPTr cells were analyzed using qRT-PCR after treatment with CdtABC. (d, e) expression of p-p53, p53, BAX, and Bcl-2 in NPTr cells was measured after CdtABC stimulation. To reveal that NPTr apoptosis was induced by *G. parasuis* CdtABC *via* a p53-dependent pathway, NPTr cells were exposed to *G. parasuis* CdtABC (500 ng/mL) with or without pifithrin-α (40 μM), a specific p53 blocker. (f) the percentage of apoptotic cells in NPTr cells was measured using flow cytometry. (g) activity of caspase-3 was measured. (H) expression of p53, BAX and Bcl-2 in NPTr cells was examined after CdtABC stimulation. Band intensity ratios were calculated from Western blot and values give relative to control cells. The statistical significance of the indicated *P* values was determined as: *P < 0.05, **P < 0.01, ***P < 0.001. All data shown are representative of at least three independent experiments.

NPTr cells were stimulated with CdtABC (0, 5, 50, or 500 ng/mL) for 36 h. The apoptotic effect of CdtABC was quantified using Annexin V-FITC/PI staining. As shown in Figure 3a, the apoptotic effect (indicated by the percentage of apoptotic cells) induced by CdtABC was significantly increased in a concentration-dependent manner. The activity of the apoptosis factor caspase-3 was determined to confirm its effect in NPTr cells. We found that CdtABC treatment enhanced the activity of caspase-3 in a concentration-dependent manner compared to untreated cells (Figure 3b). In the process of DNA damage response upon CdtABC stimulation, activated ataxia-telangiectasia-mutated (ATM) and ataxia telangiectasia and Rad3-related (ATR) proteins phosphorylate a variety of substrates, including p53. To examine the involvement of the p53 pathway in this process, the mRNA and protein levels of downstream apoptotic genes, including *p53*, *p-p53*, *BAX*, and *Bcl-2* were analysed using quantitative real-time PCR (qRT-PCR) and Western blot. Our results showed that the expression of *p-p53, p53*, and *BAX* was upregulated, whereas *Bcl-2* was downregulated in NPTr cells (Figure 3c). Moreover, the conclusions drawn by Western blot were consistent with the qRT-PCR results (Figure 3d,e). To demonstrate that p53 is directly involved in *G. parasuis* CdtABC-induced apoptosis, NPTr were exposed to *G. parasuis* CdtABC (500 ng/mL) with or without pifithrin-α (40 μM) (PFT-α), a specific p53 blocker. Flow cytometry analysis revealed that the percentage of CdtABC-induced apoptotic cells significantly decreased after pretreatment with PFT-α (Figure 3f). Furthermore, the activity of caspase-3, and the expression level of p53 and BAX also decreased, whereas the expression level of Bcl-2 increased (Figure 3g,h). These data suggest that CdtABC treatment activates the p53-dependent pathway and ultimately induces apoptosis in NPTr cells.

### VRAC blockers inhibit CDT-induced apoptosis in NPTr cells

**Figure 4.**
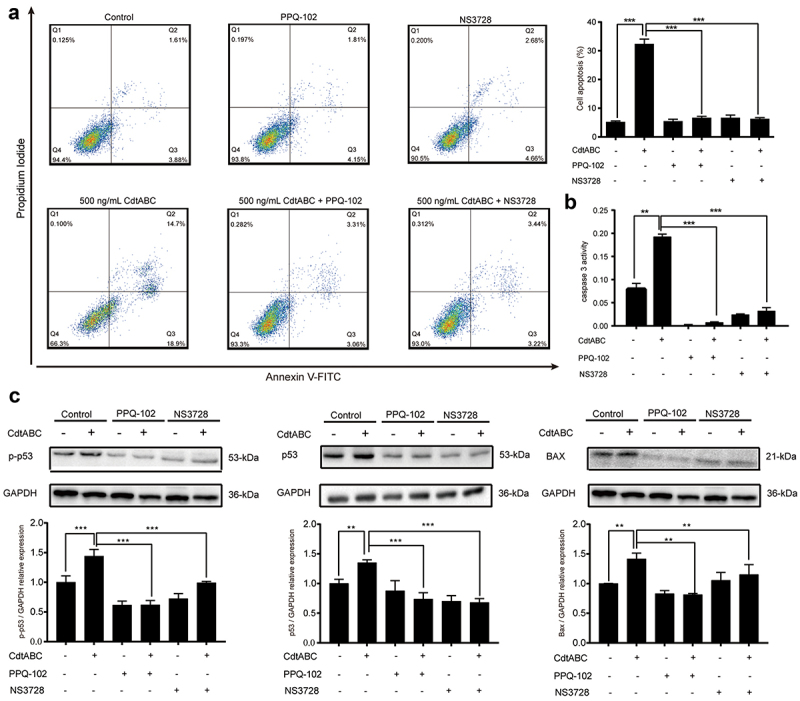
VRAC blockers inhibit *G. parasuis* CdtABC-induced apoptosis in NPTr cells. NPTr cells were pre-treated with 30 μM PPQ-102 or 10 μM NS3728 for 2 h and subsequently exposed to 500 ng/mL CdtABC for another 36 h. (a) the percentage of apoptotic cells in NPTr was measured using flow cytometry. (b) after CdtABC treatment, the activity of the apoptosis factor caspase-3 was measured. (c) the expression of p-p53, p53, and BAX protein relative to GAPDH in NPTr cells was analyzed using Western blot. Band intensity ratios were calculated from Western blot, and values are given relative to control cells. The statistical significance of the indicated *P* values was determined as: *P < 0.05, **P < 0.01, ***P < 0.001. All data shown are representative of at least three independent experiments.

Cell volume changes during cell death with isosmotic cell shrinkage, a process called AVD, is a marker of apoptosis [21]. In recent research, VRAC-mediated AVD has been shown to be related to the anti-apoptotic effect of cancer cells towards the anticancer drug cisplatin, a DNA-damaging agent, as it plays a significant role in the intrinsic apoptotic pathway, including the expression of BAX/Bcl-2 and activation of caspase-9/3/7 [37]. Thus, it is plausible to speculate a connection between CDT-induced apoptosis and VRAC-mediated AVD. Therefore, we hypothesized that VRAC is associated with CDT-triggered apoptosis. In the present study, NPTr cells were pre-treated with the VRAC blockers PPQ-102 or NS3728 for 2 h, followed by treatment with 500 ng/mL CDT. We found that treatment with either PPQ-102 or NS3728 inhibited CDT-induced apoptosis, as shown by the flow cytometry analysis of Annexin V-FITC/PI staining (Figure 4a). In addition, the presence of either PPQ-102 or NS3728 decreased the activity of apoptosis factor caspase-3 in NPTr cells (Figure 4b). To further confirm the role of VRAC, protein levels of p-p53, p53, and BAX were measured. As expected, the expression levels of p-p53, p53, and BAX markedly decreased after priming with either PPQ-102 or NS3728 in NPTr cells (Figure 4c). Together, these results indicate that the VRAC blockers PPQ-102 and NS3728 reduce CDT-induced apoptosis in NPTr cells.

### Transient knockdown of LRRC8A reduces CDT-induced apoptosis in NPTr cells

**Figure 5.**
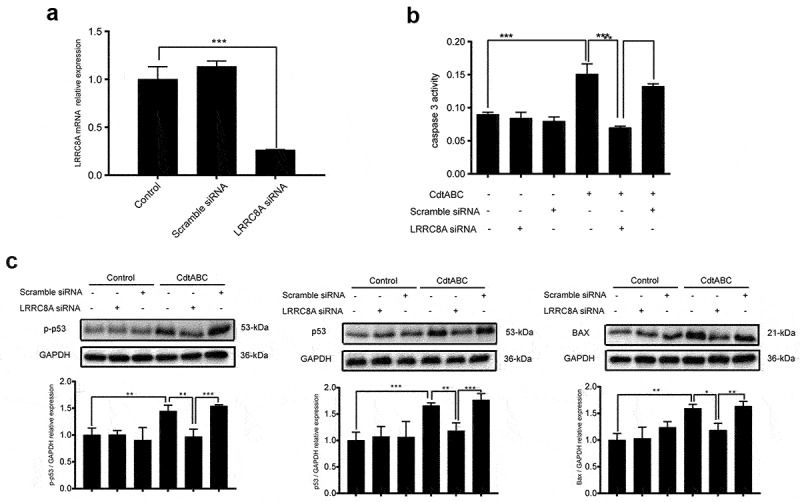
LRRC8A knockdown reduces *G. parasuis* CdtABC-induced NPTr apoptosis. NPTr cells were pre-treated with 25 nM LRRC8A siRNA or 25 nM scramble siRNA for 24 h prior to expose to 500 ng/mL CdtABC for another 36 h. (a) the interference efficiency of LRRC8A siRNA was measured using qRT-PCR. Scramble siRNA serves as a negative control. (b) the activity of apoptosis factor caspase-3 was measured in NPTr cells. (c) expression of p-p53, p53, and BAX relative to GAPDH in NPTr cells were analyzed using Western blot. Band intensity ratios were calculated from Western blot and values give relative to control cells. The statistical significance of the indicated *P* values was determined as: *P < 0.05, **P < 0.01, ***P < 0.001. All data shown are representative of at least three independent experiments.

VRAC blockers have been reported to exhibit no ability to selectively inhibit a single family of chloride channels, leading to inhibition of other anion channels [38]. LRRC8A has been identified as an essential component of the VRAC, contributing to a decrease in the regulatory volume [25]. Therefore, we employed siRNA technology to knock down the expression of *LRRC8A* (the knockdown efficiency was measured using qRT-PCR 24 h post-transfection) to explore the indispensability of LRRC8A in CDT-induced apoptosis. An analysis of *LRRC8A* mRNA levels using qRT-PCR showed that NPTr cells transfected with *LRRC8A* siRNA exhibited more than 75% reduction in the expression of *LRRC8A*, compared with the scramble siRNA transfection group, which showed no obvious difference from the control group (Figure 5a). CDT-induced apoptosis was largely attenuated in *LRRC8A*-silenced cells, as evidenced by the decreased caspase-3 activation (Figure 5b). Meanwhile, the next series of experiments in control NPTr cells revealed enhanced expression levels of p53, p-p53, and BAX after CDT stimulation. However, this increase was severely suppressed in *LRRC8A*-silenced cells (Figure 5c). These data suggest that *LRRC8A* knockdown in NPTr reduces CDT-induced apoptosis.

### Knockout of *LRRC8A* reduces CDT-induced apoptosis in NPTr cells

**Figure 6.**
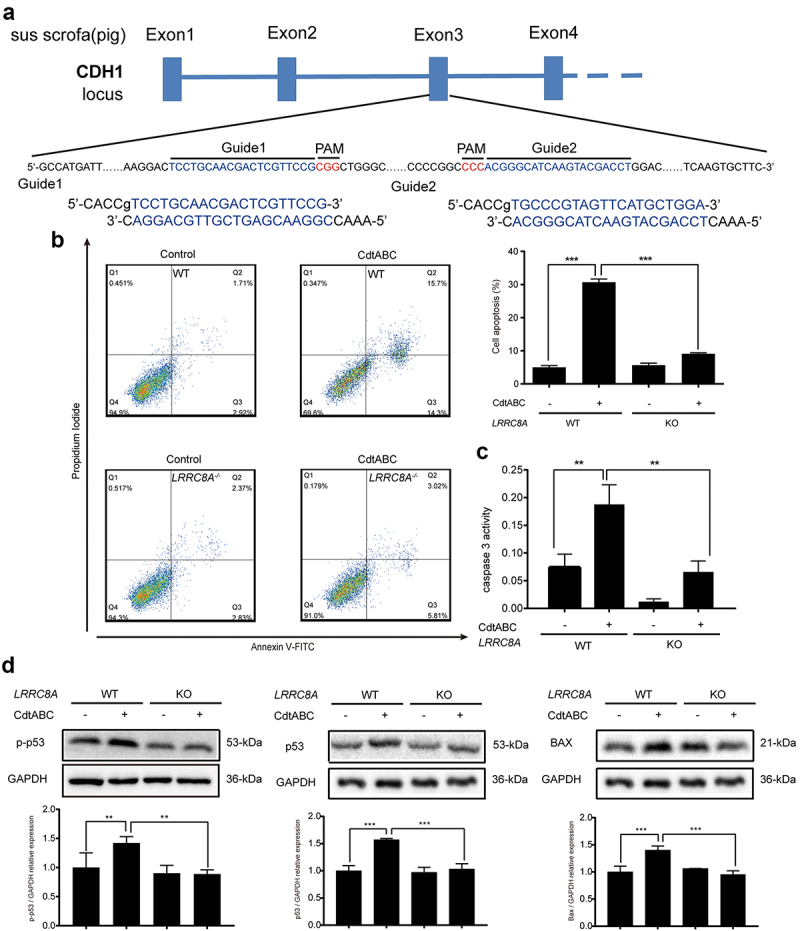
LRRC8A knockout reduces *G. parasuis* CdtABC-induced NPTr apoptosis. wild-type NPTr cells or *LRRC8A*^–/–^ NPTr cells were treated with CdtABC for 36 h. (a) *LRRC8A*^–/–^ NPTr cell line was established by CRISPR/Cas9-mediated genome editing. Two gRNAs matching the sequences flanking the exon 3 were used to achieve deletion of a large genomic fragment containing the exon 3. The upper panel shows the relevant part of LRRC8A genome structure, and the lower panel shows the sequences of the targeted region. (b) the percentage of apoptotic cells in NPTr cells was measured using flow cytometry. (c) the activity of apoptosis factor caspase-3 was measured in NPTr cells. (d) expression of p-p53, p53, and BAX relative to GAPDH in wild-type NPTr cells and *LRRC8A*^–/–^ NPTr cells were analyzed using Western blot. GAPDH expression is the loading control. The statistical significance of the indicated *P* values was determined as: *P < 0.05, **P < 0.01, ***P < 0.001. All data shown are representative of at least three independent experiments.

To rule out the potential off-target effects of siRNA, *LRRC8A* gene was knocked out in NPTr cells using CRISPR/Cas9 technology (Figure 6a). The apoptotic effect of CDT was further quantified using Annexin V-FITC/PI staining assays. In line with the results of siRNA knockdown, flow cytometry analysis revealed that knockout of *LRRC8A* resulted in a significant decrease in the percentage of cell death compared to WT cells in response to CDT (Figure 6b). Meanwhile, the activity of caspase-3, which was enhanced in wild-type cells after CDT treatment, was attenuated in *LRRC8A*^*–/–*^ cells (Figure 6c). In addition, we found that the expression of p-p53, p53, and BAX proteins was significantly reduced in *LRRC8A*^*–/–*^ NPTr cells compared to that in WT cells after CDT treatment for 36 h (Figure 6d). These data further demonstrate that the absence of *LRRC8A* reduces CDT-triggered apoptosis in NPTr cells.

### Re-expression of *sus scrofa LRRC8A* in *LRRC8A*^–/–^ *NPTr* cells restores CDT-induced apoptosis

**Figure 7.**
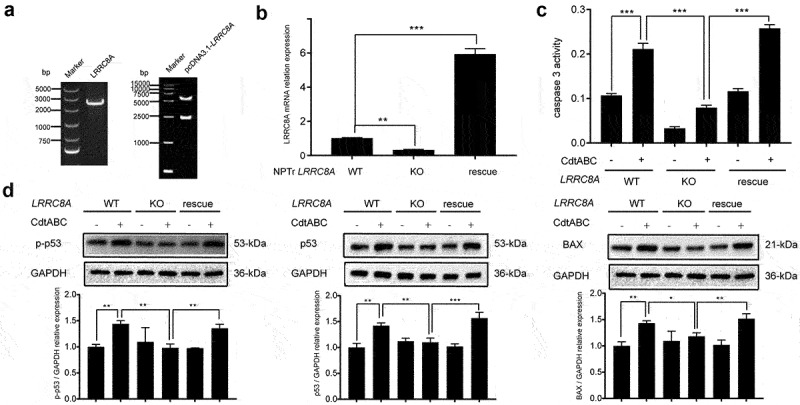
Re-expression of *Sus scrofa* LRRC8A in *LRRC8A*^–/–^ NPTr cells restores CdtABC-induced apoptosis. wild-type NPTr cells, *LRRC8A*^–/–^ NPTr cells, and LRRC8A rescued cells were treated with CdtABC for 36 h. (a) identification of LRRC8A and pcDNA3.1-LRRC8A using PCR. (b) *Sus scrofa* LRRC8A was stably expressed in the knockout cells and its expression levels were measured using qRT-PCR. (c) the activity of apoptosis factor caspase-3 was measured in wild-type NPTr cells, *LRRC8A*^–/–^ NPTr cells, and LRRC8A rescued cells. (d) expression of p-p53, p53, and BAX relative to GAPDH in wild-type NPTr cells, *LRRC8A*^–/–^ NPTr cells, and LRRC8A rescued cells. Band intensity ratios were calculated from Western blot and values give relative to control cells. The statistical significance of the indicated *P* values was determined as: *P < 0.05, **P < 0.01, ***P < 0.001. All data shown are representative of at least three independent experiments.

To further investigate the role of LRRC8A, a pcDNA3.1-LRRC8A plasmid was constructed to restore the expression of LRRC8A. A stable cell line, called “LRRC8A-RE” was established by puromycin selection (Figure 7a). The efficiency of LRRC8A rescue was analysed *via* qRT-PCR after the knockout cell lines were transfected with the plasmid. The qRT-PCR assay results indicated that *LRRC8A* mRNA levels increased by more than 6-fold compared to those in WT cells (Figure 7b). Compared with knockout cells, the activity of the apoptosis factor caspase-3 was remarkably augmented in LRRC8A-RE cells (Figure 7c). In contrast to the effect of *LRRC8A* knockout, re-expression of LRRC8A significantly enhanced the expression of p-p53, p53, and BAX (Figure 7d). Taken together, re-expression of LRRC8A in *LRRC8A*^–*/*–^ NPTr cells restore CDT-induced apoptosis in NPTr cells, which could be attributed to regaining sensitivity to CDT in *LRRC8A*^–*/*–^ NPTr cells when complemented with *Sus scrofa* LRRC8A.

## Discussion

**Figure 8.**
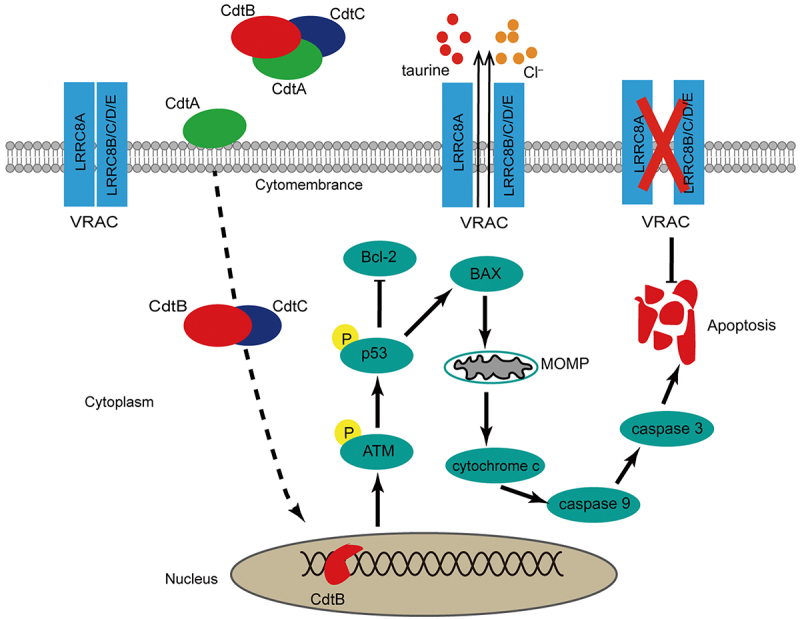
The important role of LRRC8A plays in *G. parasuis* CdtABC-induced apoptosis. the dash line indicates that CdtABC is internalized into the cell followed by the relocation of CdtB to the nucleus through an unknown pathway. As possessing DNase activity, CdtB brings about DNA double-strand breaks (DSB), which leads to DNA damage response. Induction of apoptosis *via* p53 pathway is generally elicited by CdtABC exposure as DNA damage. Activation of p53 decreased the expression of anti-apoptosis factor Bcl-2 and increased the expression of pro-apoptosis factor BAX, leading to increased mitochondrial outer-membrane permeabilization, cytochrome *c* release, activation of caspase-9 and subsequent executer caspase-3. LRRC8A is also activated by pro-apoptotic stimuli. Activation of LRRC8A triggers cell shrinkage (apoptotic volume decrease) as Cl^–^ and osmolyte efflux through CdtABC-activated VRAC, which is essential for the phosphorylation of p53 and the progression of apoptosis. Thus, LRRC8A promotes the pro-apoptosis effect of CdtABC.

The aim of this study was to explore the influence of *G. parasuis* CDT on NPTr and the role of LRRC8A in this process. Two main findings drawn from our study are as follows: (i) *G. parasuis* CDT could induce NPTr apoptosis *via* a p53-dependent pathway, suggesting that this pathogen compromises the integrity of the respiratory tract epithelium; (ii) downregulation of LRRC8A attenuates CDT-induced apoptosis, whereas re-expression of LRRC8A in *LRRC8A*^–/–^ cells effectively restores apoptosis, indicating that LRRC8A is involved in the regulation of apoptosis. These results support the model shown in Figure 8.

CDT plays a significant role in bacterial pathogenicity with the ability of invading the immune system and destroying the epithelial barrier [8,9]. CDT is composed of three subunits, CdtA, CdtB, and CdtC, with CdtB possessing DNase activity and a function that brings about a DNA damage response (DDR), which represents DNA single-strand breaks (SSB) or double-strand breaks (DSB) [39]. DDR is characterized by irreversible cell cycle arrest in the G2/M phase and eventual apoptosis [11,40]. The virulent strains of *G. parasuis* exhibit strong colonization and pathogenicity in the lungs and trachea [41]. However, the role of apoptosis in NPTr cells in response to CDT treatment remains unclear.

The p53 signalling pathway is vital for regulating the cell cycle, DNA damage, and mitochondrial pathway of apoptosis [14]. The results of the present study suggest that NPTr cells exposed to 500 ng/mL CDT showed upregulated expression of p-p53, p53, and BAX, accompanied by downregulation of Bcl-2, as well as increased activity of apoptosis factor caspase-3, implying that *G. parasuis* CDT-induced apoptosis in NPTr cells depends on the p53-mediated mitochondrial pathway. Intriguingly, recent studies have clearly demonstrated that the apoptotic pathway induced by CDT is not identical, depending on the bacterial strain and its target cells, although they exhibit similar activity to DNase. For example, CDT from *H. ducreyi* induces p53-independent apoptosis in ATM-deficient lymphoblasts [8], whereas apoptosis in pulmonary alveolar macrophage (PAM) cells and porcine kidney epithelial (PK-15) cells induced by *H. parasuis* CDT is p53-dependent [42]. A plausible explanation for this discrepancy may be that the role of p53 in CDT-induced apoptosis depends on the specific cell type and source of the toxin [43].

To the best of our knowledge, a major hallmark of apoptosis is normotonic shrinkage of cells, also known as apoptotic volume decrease (AVD), which is considered an early prerequisite for apoptotic events and precedes other apoptotic changes, such as caspase activation [20,21]. AVD is a dynamic process that is divided into AVD_1_, AVD_T_, and AVD_2_, which reflects a balance between intra- and extracellular osmotic pressure between pro-apoptotic and anti-apoptotic processes [21]. In recent research, VRAC was shown to be required for AVD, especially in the AVD_1_ stage [44]. Inhibition of VRAC alleviates AVD and ultimately suppresses apoptosis [21]. As a core component of VRAC, LRRC8A participates in the regulation of chemotherapy resistance by influencing its anti-apoptotic activity. For example, Sørensen et al. [37] demonstrated that LRRC8A is associated with sensitivity to the anti-tumour drug cisplatin in several cell lines *via* affecting the AVD process and p53 activation. According to genome sequence alignment, *Sus scrofa* LRRC8A shares more than 99.34% amino acid sequence similarity with *Homo sapiens* and *Mus musculus*. Considering the high structural conservation of LRRC8A and the similarity of pro-apoptotic mechanisms between anti-tumour drugs and CDT, it is plausible to postulate that the function of LRRC8A might also be involved in CDT-induced apoptosis in NPTr cells. Therefore, we explored the functional significance of LRRC8A in CDT-induced apoptosis in NPTr cells. We demonstrated that inhibition of VRAC with either PPQ-102 or NS3728 in NPTr cells markedly reduced CDT-induced apoptosis *via* the p53/BAX/caspase-3 pathway. As the chemical blocker of VRAC usually inhibits other channels, attenuated apoptosis was confirmed by siRNA-mediated knockdown and CRISPR/Cas9-mediated LRRC8A deletion in NPTr cells. Conversely, re-expression of LRRC8A in *LRRC8A*^–/–^ NPTr cells re-established their sensitivity towards CDT, which confirms our conclusion to a high degree that CDT-induced apoptosis is correlated with the expression of LRRC8A. Notably, the effects of LRRC8A on different cell lines could be adverse. For example, LRRC8A is highly expressed in hepatocellular carcinoma tissues, which inhibits apoptosis and strengthens cell proliferation and migration while promoting apoptosis in glioblastoma, ovarian, and alveolar carcinoma cells [31,37,45]. The reason why LRRC8A plays opposing roles in different cell lines is unknown. A possible explanation may be that LRRC8A not only serves as a part of VRAC but also as a signal protein that regulates apoptosis by different mechanisms. Nevertheless, there are still some limitations to our study that require further investigation. First, although LRRC8A is involved in this process, its exact molecular mechanism remains unclear. On the one hand, activation of LRRC8A-containing transporter (VRAC) might contribute to AVD and p53 activation. On the other hand, LRRC8A has also been shown to be correlated with some signalling pathways, such as PKCα-LRRC8A-PI3K/AKT-p21 [45], which may be the underlying mechanism of LRRC8A in apoptosis as well. Second, VRAC can be activated not only by cell swelling or reduced intracellular ionic strength but also by other pro-apoptotic stimuli in the absence of cell swelling [46,47]. In the present study, we did not provide direct evidence for assessing VRAC activation in response to CDT. Additionally, to date, the researches for LRRC8A in apoptosis are focused on the mitochondria-dependent pathway. A myriad of experimental studies are still required to elucidate the exact mechanism and its role in the exogenous apoptotic pathway.

## Conclusion

Our research reveals that *G. parasuis* CDT induces apoptosis in NPTr cells by activating the p53 signalling pathway. Furthermore, LRRC8A, the core component of VRAC, is a crucial regulator of this process. Its downregulation and knockout reduce CDT-induced apoptosis in NPTr cells. Our findings imply that LRRC8A plays a central role CDT-induced apoptosis in *G. parasuis* and provide a light on its potential as an apoptosis effector or biomarker.

## Data Availability

The datasets generated for this study are available on request to the corresponding author.
